# Lignins: Biosynthesis and Biological Functions in Plants

**DOI:** 10.3390/ijms19020335

**Published:** 2018-01-24

**Authors:** Qingquan Liu, Le Luo, Luqing Zheng

**Affiliations:** 1 College of Life Sciences, Nanjing Agricultural University, Nanjing 210095, China; liuqingquan@cnbg.net; 2 Institute of Botany, Jiangsu Province and Chinese Academy of Sciences, Nanjing 210014, China; 3 College of Resources and Environmental Sciences, Nanjing Agricultural University, Nanjing 210095, China; luole@njau.edu.cn

**Keywords:** lignin, genetic modification, lodging resistance, diseases resistance, stress tolerance

## Abstract

Lignin is one of the main components of plant cell wall and it is a natural phenolic polymer with high molecular weight, complex composition and structure. Lignin biosynthesis extensively contributes to plant growth, tissue/organ development, lodging resistance and the responses to a variety of biotic and abiotic stresses. In the present review, we systematically introduce the biosynthesis of lignin and its regulation by genetic modification and summarize the main biological functions of lignin in plants and their applications. We hope this review will give an in-depth understanding of the important roles of lignin biosynthesis in various plants’ biological processes and provide a theoretical basis for the genetic improvement of lignin content and composition in energy plants and crops.

## 1. Introduction

**Figure 1:**
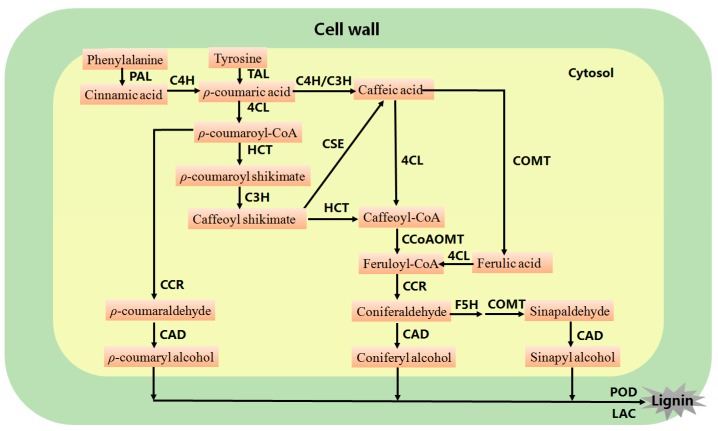
The general biosynthesis pathway of lignin in higher plants. PAL, phenylalanine ammonia-lyase; TAL, tyrosine ammonia-lyase; C4H, cinnamate 4-hydroxylase; 4CL, 4-coumarate: CoA ligase; CCR, cinnamoyl-CoA reductase; HCT, hydroxycinnamoyl-CoA shikimate/Quinatehydroxycinnamoyltransferase; C3H, *p*-coumarate 3-hydroxylase; CCoAOMT, caffeoyl-CoA *O*-methyltransferase; F5H, ferulate 5-hydroxylase; CSE, caffeoyl shikimate esterase; COMT, caffeic acid *O*-methyltransferase; CAD, cinnamyl alcohol dehydrogenase; LAC, laccase; POD, peroxidase.

Lignin is one of the most important secondary metabolite which is produced by the phenylalanine/tyrosine metabolic pathway in plant cells. It is the second most profuse biopolymers that accounts for 30% of the organic carbon content in biosphere [1]. Lignin biosynthesis is a very complex network that is divided into three processes: (i) biosynthesis of lignin monomers, (ii) transport and (iii) polymerization (Figure 1). After a series of steps involving deamination, hydroxylation, methylation and reduction, lignin monomers are produced in cytoplasm and transported to the apoplast. Finally, lignin is generally polymerized with three main types of monolignols (sinapyl alcohol, S unit; coniferyl alcohol, G unit and *p*-coumaryl alcohol, H unit) by peroxidase (POD) and laccase (LAC) in secondary cell wall [2,3,4,5]. In addition, several other compounds including hydroxycinnamaldehydes, tricin flavones, hydroxystilbenes and xenobiotics etc. have also been recognized to be lignin subunits [6,7,8,9,10,11,12]. In differentiating protoxylem tracheary elements of *Arabidopsis*, lignin monomers can be free to diffuse in the extracellular space but are only polymerized in the secondary cell walls [13].

Lignin and its related metabolism play important roles in the growth and development of plants. As a complex phenolic polymer, lignin enhances plant cell wall rigidity, hydrophobic properties and promotes minerals transport through the vascular bundles in plant [13]. In addition, lignin is an important barrier that protects against pests and pathogens [14]. Lignin metabolism can also be actively involved in plant lodging resistance and in response to various environmental stresses [15,16,17,18,19].

As one of the important components of plant cell wall, lignin is of great significance to plant growth and environmental adaptability. In addition, lignin itself can also be used as a resource for the field of energy or pharmaceutical industry. The study of lignin biosynthesis and its function will have a significant impact on industrial, agricultural production and other human activities. Therefore, we briefly summarize the biosynthesis, function and application of lignin to provide a mini review of the research on lignin.

## 2. Genetic Modification of Lignin Biosynthesis

Lignin content, composition and structure can be altered using genetic modification. The effects of single or multiple lignin biosynthesis gene expression on phenotypic traits of transgenic plants can be directly observed and identified (Table 1). In the past two decades, many researchers have used lignin engineering to modify lignin content and composition in plants [20]. Lignin content in *Arabidopsis thaliana* quadruple mutant (*pal1*/*pal2*/*pal3*/*pal4*) of *PAL* genes, decreased by 20–25% as compared with the wild type, while the quadruple mutant also displayed reduced levels of salicylic acid and increased susceptibility to pathogen [21]. Gui et al. [22] found that the inhibition of *Os4CL3* gene expression significantly reduced the lignin content and height of rice plants. Suppression of the mRNA level of *4CL* gene in *Pinus radiata* reduced the lignin content of transgenic plants by 36–50%, which mainly due to depletion of guaiacyl lignin and resulted in a dwarfed phenotype of plant [23]. Cinnamoyl-CoA reductase (CCR) and cinnamyl alcohol dehydrogenase (CAD) are the last two enzymes that function in the monolignols synthesis pathway, while the disruption of their function also affects the lignin content as found in the *Arabidopsis thaliana ccc* triple mutant (*cad c cad d ccr1*), i.e., lignin content was reduced by 50% as compared with the wild type and was accompanied by the male-sterile phenotype [24]. Recently, Van Acker et al. [25] found that CAD1-deficiency resulted in different metabolic routes for coniferaldehyde and sinapaldehyde and modified lignin content and structure in poplar. In C4 forage grasses, lignin deposition in the thick-walled parenchyma bundle-sheath cells affects digestibility of forage by animals, down-regulation of *CCR* gene is considered to be an effective strategy for the production of low-lignin C4 *Paspalumdilatatum* [26]. Hydroxycinnamoyl: CoA transferase (HCT), *p*-coumarate 3-hydroxylase (C3H) and caffeate/5-hydroxyconiferaldehyde *O*-methyltransferase (COMT) are involved in the synthesis of sinapyl alcohol (S unit) and coniferyl alcohol (G unit). Interference of the expression of *CCR1* and *COMT1* genes significantly altered lignin content and composition in ryegrass and enhanced digestibility without significant negative effects on either plant fitness or biomass production [27]. Shadle et al. [17] showed that transgenic *Medicago sativa*, expressing an HCT antisense construct, led to a significant decrease in lignin content and an obvious change in the lignin composition, while exhibited obvious stunting, decreased biomass and delayed flowering. Vanholme et al. [28] found that *Populus nigra hct1* mutant had a modified H lignin disposition. Inhibition of *C3H* expression in poplars also reduced the lignin content of poplar and changed its S/G ratio [29]. Zhang et al. [30] reported that overexpression of the monolignol 4-*O*-methyltransferase *MOMT4* gene resulted in a 24% reduction in lignin content in *Arabidopsis* cells and increased saccharification yields of transgenic plants. Ferulate 5-hydroxylase (F5H) is one of key enzymes that regulate S/G lignin composition in plants, recent study has shown that the heterologous expression of angiosperm F5H induced ectopic productions of S lignin in the gymnosperm cell walls [31]. In rice, overexpression of a F5H gene *OsCAld5H1* increased the content of S units, while down-regulation of it enhanced the production of G lignin [32]. Recently, caffeoyl shikimate esterase (CSE) was reported to be involved in lignin biosynthesis in *Arabidopsis thaliana*, *Medicago truncatula* and hybrid poplar [33,34,35]. In addition, tricin was shown to react with monolignols, and down-regulated chalcone synthase (CHS) significantly reduced the contents of apigenin- and tricin-related flavonoids, resulting in a strongly reduced tricin lignin [11]. Eudes et al. [36] found that *Sorghum bicolor* (SbCOMT) can methylate the tricin precursors (luteolin and selgin) and be involved in the biosynthesis of lignin-linked tricin and S lignin units in sorghum.

After the synthesis of monomers in the cytoplasm, they are transported across the cell membrane and then polymerized in the cell wall [3]. *Arabidopsis AtABCG29* knockout mutants exhibited less lignin content and more sensitivity to *p*-coumaryl alcohol, due to *p*-coumaryl alcohol transport activity of AtABCG29 protein [2]. Peroxidases and laccases are two key enzymes that participate in the polymerization of monomers [40,46]. Overexpression of POD increases the content of phenol and lignin in plants [47]. Shigeto et al. [48] found that *AtPrx* double mutants (*atprx2*/*atprx25*, *atprx2*/*atprx71* and *atprx25*/*atprx71*) had lower lignin content than single mutants but did not seriously affect the growth. Recently, a new class III peroxidase PRX17 (At2g22420) in *Arabidopsis thaliana* was reported to participate in lignin biosynthesis and be regulated by a MADS-box transcription factor AGL15 [49]. Compared to above mentioned lignin biosynthesis genes, the role of *laccase* in lignin biosynthesis is relatively poorly studied in the past years. Wang et al. [50] confirmed that the overexpression of cotton laccase gene significantly increased the total lignin content of transgenic poplar. Berthet et al. [46] found that lignin content in the stems of two *Arabidopsis* laccase double mutants, *lac4-1 lac17* and *lac4-2 lac17*, decreased by 20% and 40%, respectively. Surprisingly, lignin deposition in roots has almost completely disappeared in the *Arabidopsis lac11 lac4 lac17* triple mutant [40]. Recently, a new lignification-related LACCASE5 was identified in *Brachypodium distachyon*, which lead to a modification on lignin content and composition [51].

Studies have shown that phenylpropanoid metabolism and lignin-specific synthesis can be regulated by transcription factors. The most widely studied transcription factor for lignin biosynthesis is the MYB (v-myb avian myeloblastosis viral oncogene homolog) family. It has been found that overexpression of *Arabidopsis thaliana* MYB58 and MYB63 transcription factors activated the expression of lignin biosynthesis-related genes and promoted the lignification of cells. While the other transcription factors, such as *EgMYB1* from eucalyptus, *MusaMYB31* from *Musa* inhibited the expression of genes related to lignin biosynthesis and negatively regulated lignin accumulation [52,53,54]. In addition to the MYB transcription factor, some members of NAC transcription factor family in *Arabidopsis* were able to control lignin biosynthesis by regulating the entire cell wall synthesis-related genes [54]. WRKY transcription factors can also work as regulators of lignin biosynthesis genes. Down-regulation of a WRKY transcription factor exhibited increased lignin level and enhanced biomass yield in *Medicago sativa* L. [55]. In the last few years, more and more lignin metabolism related genes have been identified by in vivo experiments, such as maize Caffeoyl-CoA *O*-methyl transferase gene *CCoAOMT1*, switchgrass *UDP-Arabinomutase* gene, *Betula platyphylla* MADS-box gene *BpMADS12* and *Eriobotrya japonica* heat shock factors *EjHSF3* [42,43,56,57]. In addition to the use of genetic engineering technology to change lignin content and composition; the growth, development and environmental adaptability of transgenic plants should be further studied.

## 3. Role of Lignin in Plant Growth and Development

As one of the main components of the plant cell wall, lignin confers to the function of multiple types of cells in plants tissues and organs [58]. Lignin metabolism is involved in plant growth and development, interference of lignin biosynthesis, especially H units, often leading to inhibition of plant growth and deformity development [59]. In some plants, lignin accumulation is important for the seed propagation [60]. The deposition of lignin in seed coat can protect the seeds from external adverse factors. Liang et al. [61] found that the lower content of lignin in *Arabidopsis thaliana* mutant seed coat significantly decreased the seed germination rate, in comparison to wild type. The content of lignin in the *Arabidopsis thaliana CCR1* mutant was significantly reduced, accompanied by stunted growth and reduced number of seeds [18,20]. In *Arabidopsis thalianaC4H* mutant, the growth and lignin accumulation was inhibited, apical dominance was lost and showed male sterility [62]. Similarly, simultaneous disruption of *CAD* and *CCR* leading to an obvious decrease in the content of lignin in *Arabidopsis thaliana* and a change in lignin composition, resulting in severe suppression of plant growth and male sterility, which may be related to lack of lignin in the anther [24]. Herrero et al. [39] found that in comparison with the wild type, total lignin and S unit contents were obviously decreased in *Arabidopsis thaliana* peroxidase *AtPrx72* knockout mutant, which had slow growth, less branches, smaller flower and stem than the wild-type plants, as well as significantly reduced photosynthetic efficiency. However, the mechanisms of lignin affecting plant growth and development are poorly understood. Recently, Bonawitz et al. [59] have suggested that the transcriptional process and signaling pathways responding to cell wall defects may play an important role in lignin-deficient induced stunted growth.

## 4. Role of Lignin in Plant Lodging Resistance

Lodging resistance can prevent plant stems from bending or breaking, it is one of the most important traits that affect crop growth and grain yield [63]. Numerous studies have shown that the lodging resistance of crops is related to plant height, biomass, stem diameter and the composition and characteristics of stem cell walls [64,65,66]. Lignin accumulation in cell wall significantly enhance the mechanical strength of plant stalks. It has important implications for crop lodging resistance [15].

Peng et al. [67] added exogenous paclobutrazol to lodging-resistant/susceptible cultivar of winter wheat and found that paclobutrazol significantly reduced the internode length of wheat, promoted the lateral growth and increased lignin deposition, the activities of lignin biosynthesis enzymes and thickness of internode, thus improved the wheat lodging tolerance. Another study reported that the crop density can significantly change the morphological characteristics and the lignin biosynthesis of the stem and thus enhance the mechanical strength of the stem and reduce the hazard of lodging [68]. Hu et al. [69] reported that the lignin content and lignin biosynthesis enzymes (PAL, 4CL, CAD and POD) activities had important roles in the lodging resistance according to the analysis of lignin metabolism related indexes in *Fagopyrum esculentum* Moench varieties with a different lodging resistance. 

In addition, nutrient elements have an important effect on plant lignin biosynthesis and lodging resistance. Silicon can enhance the expression of rice *CAD* gene, improve the accumulation of lignin and increase the strength of the stalk, thereby enhancing the lodging resistance [70]. However, excessive nitrogen fertilizer significantly decreased the mechanical strength of stalk and the lodging resistance by reducing lignin biosynthesis in buckwheat, rapeseed and japonica rice [71,72,73]. Kong et al. [74] found that the addition of K^+^ could significantly alleviate the effect of high NH_4_^+^ on the wheat culm strength. Therefore, it was suggested that the decrease of lodging resistance induced by nitrogen fertilizer was possibly related to the inhibition of uptake of K^+^ which increased the lignin accumulation in the vascular bundles.

## 5. Relationship between Lignin Biosynthesis and Plant Stress Adaptation

Plant cell wall is the first barrier against external hazards, one of the general reactions of plants under biotic and abiotic stresses is the accumulation of reactive oxygen species, accompanied by an increase in lignin accumulation [19,75,76]. Therefore, lignin metabolism has a certain relevance with plant disease resistance, insect resistance, the tolerance of drought, salt, heat, cold, heavy metals and other stresses [19].

### 5.1. Roles of Lignin Biosynthesis in Plant Insect Pests and Diseases Resistance

Lignin accumulation plays an important role in the process of plant resistance to insect pests [77,78]. Rice *PAL*, *C4H* and pathogenesis-related 9 (*PR9*) genes that associated with lignin biosynthesis were significantly up-regulated in brown plant hopper-infested insect-resistant rice varieties, suggesting that these may synergistically participate in lignin biosynthesis which regulate the insect resistance of rice [79,80]. Wang et al. [81] found that an aphid penetration-induced transcription factor CmMYB19 in chrysanthemum enhanced the expression of lignin biosynthesis genes and lignin accumulation, which limited the invasion of aphids and increased chrysanthemum aphid tolerance. Fujimoto et al. [82] found that sclareol, an antimicrobial molecule, enhanced *Arabidopsis thaliana* root-knot nematode resistance by mediating the ethylene-dependent lignin accumulation in the roots. An insect-specific toxin peptide LqhIT2 enhanced the lignin content mediated by jasmonate and improved the leafrollers’ resistance to rice [83]. In summary, lignin can be used as a barrier directly or through the associated hormone signal pathway to increase insect resistance of plants.

When plants are infected with pathogens, cell wall will accumulate a large amount of lignin, in which the content of H unit is relatively higher [84,85]. Increased accumulation of lignin can provide a basic barrier against pathogen spread and reduce the infiltration of fungal enzymes and toxins into plant cell walls; Lignin-related compounds may cause fungi to lose the activity of infecting the host and prevent pathogen multiplication and movement [78,86,87]. Mandal et al. [88] found that the content of lignin in pathogenic bacteria-resistant tomato varieties was obviously higher than that of susceptible cultivars. The expression of lignin biosynthesis gene is closely related to the disease resistance in plants. *Arabidopsis CAD5* was highly expressed in roots with strong lignification and induced by pathogens invading *Arabidopsis thaliana* [45]. Cotton dirigent-like gene (*DIR*) was involved in the lignin deposition, overexpression of *GhDIR1* significantly promoted the degree of lignification and enhanced *Verticillium dahliae* resistance in transgenic plants [89]. Two *HCT* genes were identified in maize and regulated plant disease resistance by binding to NLR Rpl protein, which enhanced the expression of lignin biosynthesis pathway gene and lignin accumulation [90]. Overexpression of rice 4CL gene *OsAAE3* decreased lignin accumulation and increased sensitivity to rice blast and this may be related to the decrease of POD activity and expression of pathogen-related 1a (PR1) [37]. In maize, a CCoAOMT gene *ZmCCoAOMT2* was found to be associated with resistance to multiple pathogens, might be involved in the biosynthesis of lignin and other phenylpropanoid metabolites and regulation of programmed cell death [38].

### 5.2. Role of Lignin Deposition in Plant Heavy Metal Tolerance

As the first entry barrier for metal ion, cell wall is actively involved in the absorption and transport of heavy metals and the plant response to heavy metal stress [91,92]. Heavy metals stress can stimulate phenolic secondary metabolic synthesis pathway and increase the lignin content in secondary cell wall, thereby enhance the thickness of the cell wall. As lignin polymer contains a large number of functional groups (hydroxyl, carboxyl, methoxyl, etc.), it can bind multiple heavy metal ions (Cu^2+^, Cd^2+^, Pb^2+^, etc.) [93,94] and reduce the entry of heavy metals into the cytoplasm [95,96].

In acidic soil, Aluminum (Al) toxicity is one of the main factors that inhibit plant growth. A typical symptom of Al toxicity is the inhibition of root growth [97]. Tea plant is known for high Al-resistance and high concentrations of Al can stimulate its growth [98]. Ghanati et al. [99] found that the activities of phenylalanine ammonia-lyase (PAL) and cell wall POD in lignin biosynthesis pathway in tea plants were significantly decreased under high concentration of Al. The content of lignin was also reduced, which may be the reason of the thriving existence of tea in high concentration of Al. Another study showed that Al can induce the expression of the enzyme genes in the lignin biosynthesis pathway, the expression of *4CL*, *CAD*, *C3H* and *PAL* genes were increased under Al stress [100]. Other heavy metals such as copper (Cu), cadmium (Cd), zinc (Zn) and manganese (Mn) stress can also increase lignin content in some plants [76,96,101,102,103,104]. Increasing of Cu concentration in the culture medium enhanced the activity of PAL, CAD, caffeic acid POD, the accumulation of phenolic compounds and lignin in the suspension cells of ginseng root [105]. Bhuiyan et al. [95] found that under Cd treatment, the growth of soybean roots was inhibited, the content of lignin was increased and accompanied by the increase of POD and LAC activity and the expression of *POD* gene related to lignin biosynthesis was also up-regulated.

Lignin biosynthesis is also closely related to plant heavy metals absorption, transport and tolerance. The deposition of lignin in the cell wall of the root endoderm may inhibit the transport of heavy metal elements into the xylem or outward from the vascular bundle [106,107,108,109]. The expression level of high Zn-induced lignin biosynthesis genes in Zn/Cd hyperaccumulator *Thlaspi caerulescens* roots were higher than that in *Arabidopsis* roots; tissue sections showed that the lignin accumulation in the endodermis cell wall of *Thlaspi caerulescens* roots was significantly higher than that of the *Arabidopsis* [103]. The results indicated lignin biosynthesis was crucial for the hyperaccumulation of Zn/Cd in *Thlaspi caerulescens* and it might be related to ion absorption and transport. Our previous study found that the deposition of lignin reduced the toxic effects of Mn, which is an important mechanism of manganese tolerance in a Mn-hyperaccumulator *Phytolacca americana* [101]. Lignification of xylem in roots can reduce the transport of Cd to the shoots [110]. Recently, our results showed that Cu stress could increase the lignin accumulation in the roots of rice by enhancing the lignin polymerization. Cu stress induced hydrogen peroxide (H_2_O_2_) was involved in the regulation of lignin monomer polymerization and lignin accumulation, this process may affect the transport of Cu from the root to the shoot in rice seedling [43].

### 5.3. Role of Lignin in Plant Drought and Salt Stress Tolerance

Extreme drought and high salt stress usually occur simultaneously and induce osmotic stress that causes plant cells to lose water or even die, significantly affecting plant growth and development and resulting in serious losses of crop yield [111,112]. Lignin can reduce plant cell wall water penetration and transpiration, which helps to maintain cell osmotic balance and protective membrane integrity [113].

Many studies have shown that lignin biosynthesis is enhanced under drought stress. When suffering from drought stimulation, the content of lignin was significantly increased in the stem basal zone of *Eucalyptus urograndis* and the stem apical zone of *Eucalyptus globulus* [114]. Under drought stress, the expression levels of lignin biosynthesis related genes (*CAD* and *COMT*) and lignin content in leaves of inbred maize lines were significantly positively correlated with their drought tolerance [115]. *CCR* is a key gene in lignin monomer biosynthesis pathway, under the drought stress condition, *CCR1/2* were significantly up-regulated in the root elongation region of maize [116]. In addition, Srivastava et al. [117] found that drought stress significantly increased lignin content and CCR protein expression in developing stem of *Leucaena* seedlings and suggested that CCR-catalyzed lignin biosynthesis may play an important role in drought stress tolerance of *Leucaena.* Under drought stress, the expression of *CCoAOMT* was obviously enhanced in the root elongation region of soybean and the accumulation of lignin in the region was also increased significantly, the increase in lignin content reduced the elongation of the root cell wall and may limit the loss of water from the roots and promote the transport of water into the elongated zone [118].Recent studies have shown that lignin deposition is likely to be involved in plant salt tolerance. Overexpression of *Ipomoea batatas IbLEA14* genes enhanced transgenic callus salt and osmotic stress tolerance by increasing lignin accumulation [119]. H_2_O_2_ is one of the stresses-induced reactive oxygen species (ROS), it also plays an important regulatory role in lignin biosynthesis [76,120]. Shafi et al. [44] found that overexpression of the *Potentilla atrosanguinea* superoxide dismutase (*SOD)* and *Rheum australe* ascorbate peroxidase (*APX*) gene enhanced the tolerance of transgenic *Arabidopsis thaliana* by increasing the accumulation of lignin via maintaining the level of hydrogen peroxide. MYBs have important roles in both secondary cell wall biosynthesis and abiotic stress tolerance, overexpression of *Betula platyphylla BplMYB46* increased salt and osmotic stress tolerance and enhanced lignin deposition in transgenic birch, indicated that the abiotic stress response and lignin biosynthesis pathways may have a crosstalk with each other [41]. Therefore, as a waterproof lignin-rich barrier, lignified cell wall conferring plants tolerance to the drought and high osmotic environment.

### 5.4. Role of Lignin in Plant Temperature Stress Adaptability

Climate change affects the temperature of plant growth environment, high temperature induces the damage of biomacromolecules such as protein and nucleic acid, exacerbates membrane lipid peroxidation and disturbs the normal plant metabolism [121,122]. In contrast, low temperature stress also causes cell membrane damage, reduces plant photosynthesis and respiration and severely inhibits plant growth and development [123]. The content of lignin in plant tissues was significantly increased in the process of cold acclimation, the expression of *C3H* gene in the cold acclimated *Rhododendron* leaves was notably higher than that in the control leaves, *C3H* may affect the cell wall rigidity and water permeability by modifying S/G ratio and participate in the *Rhododendron* cold resistance [124]. In addition, heat and low temperature conditioning (LTC) can reduce low temperature-induced lignification in loquat fruit, analysis showed that LTC downregulated the expression of a HSF gene *EjHSF3*, which was involved in fruit lignification via interacted with lignin biosynthetic regulator EjAP2-1 [43].

Gindl et al. [125] found that lignin deposition in the terminal latewood tracheid of Norway spruce was positively correlated with the temperature of the periods between early September and late October. Yun et al. [126] found that postharvest high temperature-induced lignin deposition in *Satsuma mandarin* pericarp involved in resistance to environmental stress. Lignin content in *Medicago truncatula CAD1* mutant was significantly lower than in wild type and there was no significant growth difference between the wild type and mutants at normal temperature (22 °C). However, at 30 °C, the *MtCAD1* mutant growth was significantly inhibited and the growth phenotype of compensated lines were similar to the wild type. Possible reason is that reduced-lignin accumulation lead to vascular damage, thereby affecting the transpiration and causing overheating in the plant body [127]. 

## 6. Different Types of Lignin and Its Application

As a cheap, renewable resource, plant lignin is mostly used as an energy substance, or to develop new materials, e.g., lignin-based carbon fibers, due to the presence of phenolic hydroxyl groups and aliphatic hydroxyl groups in lignin structures. Lignin can be generally divided into three types according to the different plant species: softwood, hardwood and grass lignin. Softwood lignin consists exclusively of coniferyl alcohol, hardwood lignin consists mainly of coniferyl alcohol and sinapyl alcohol, grass lignin has three types of monomers (coniferyl, sinapyl and *p*-coumaryl alcohol) [128]. In addition, the non-conventional types such as the caffeyl lignin (C-lignin) were found in the seeds of vanilla orchid and several species of the Cactaceae [129].

The composition of the monomers has an important influence on the molecular structure of lignin, such as branching of the polymer and the degree of crosslinking with the polysaccharide [130]. Therefore, the monomer composition determines the degradability of lignin and the workability of lignocellulosic biomass [131,132]. For example, corn and flax lignin generally contain high content of aliphatic OH groups and non-methoxylate phenolic groups respectively, which are suitable for production of phenolic resins and polyurethane synthesis, respectively; the content of OH groups is balanced in triticale lignin, which is appropriate for polyester synthesis [133]. In addition, low molecular weight monomers derived from H and G units have a certain antioxidant capacity, making lignin also has some biological activity, such as anti-tumor [134,135].

## 7. Conclusions and Remarks

Lignin is an important organic polymer which is abundant in cell walls of some specific cells. It has many biological functions such as water transport, mechanical support and resistance to various stresses. Most of the current researches on lignin in plants are focused on the regulation of lignin content through molecular biology and molecular genetics. Reducing the accumulation of lignin in energy plants can improve the production efficiency of biofuels. However, as shown above, the reduction of lignin biosynthesis can seriously affect plant growth and development, increase the risk of crop lodging, reduce plant resistance to external biotic and abiotic stresses and thus result in a serious threat to crop production.

In the future, we should carefully consider the possible consequence of reducing the lignin content of the specific energy plant through the genetic modification of lignin biosynthesis genes, to maintain the normal plant physiological metabolism and improve the bioenergy efficiency, simultaneously. For some important crops, we could increase its lignin accumulation by genetic engineering, therefore enhance its resistance to external environmental factors and improve crop yields. In addition, modifying the composition and the proportion of monomers by regulating of specific lignin biosynthesis genes can provides an effective way to improve the application of lignin.

## Figures and Tables

**Table 1:** The function of typical lignin biosynthesis-related genes reported in the last 5 years.

Genes	Plant Species	Functions	References
*4CL*	*Oryza sativa* (*OsAAE3*)	Decreased lignin accumulation and increased sensitivity to rice blast	[37]
*CCR*	*Paspalum dilatatum* (*PdCCR1*)	Increased lignin content and altered lignin deposition	[26]
*CAD*	*Populus trichocarpa* (*PtrCAD1*)	Modified lignin content and structure	[25]
*MOMT*	*Arabidopsis thaliana* (*MOMT4*)	Depressed lignin biosynthesis and increased saccharification yields	[30]
*COMT*	*Sorghum bicolor* (*SbCOMT*)	Methylate the tricin precursors and participated in S lignin biosynthesis	[36]
*CSE*	*Populus tremula × P. alba* (*PtxaCSE1/2*)	Participated in lignin biosynthesis and affected saccharification	[35]
*CCoAOMT*	*Zea mays* (*ZmCCoAOMT2*)	Participated in lignin biosynthesis and resistance to multiple pathogens	[38]
*HCT*	*Populus nigra* (*HCT1*)	Modified H lignin disposition	[28]
*F5H*	*Oryza sativa* (*OsCAld5H1*)	Increased the content of S units, decreased G lignin	[31]
*POD*	*Arabidopsis thaliana* (*AtPrx72*)	Participated in lignin biosynthesis, growth and development of plant	[39]
*LAC*	*Arabidopsis thaliana* (*LAC11*)	Participated in lignin biosynthesis and the development of root, anthers and vascular	[40]
*ABC*	*Arabidopsis thaliana* (*AtABCG29*)	Participated in *p*-coumaryl alcohol transport	[2]
*MYB*	*Betula platyphylla* (*BplMYB46*)	Increased salt/osmotic stress tolerance and enhanced lignin deposition	[41]
*MADS*	*Betula platyphylla* (*BpMADS12*)	Participated in lignin biosynthesis and regulation of brassinosteroid signaling pathway	[42]
*HSF*	*Eriobotrya japonica* (*EjHSF3*)	Participated in fruit lignification	[43]
*SOD*	*Potentilla atrosanguinea* (*SOD*)	Enhanced lignin accumulation and stress tolerance of *Arabidopsis*	[44]
*APX*	*Rheum australe* (*APX)*	Enhanced lignin accumulation and stress tolerance of *Arabidopsis*	[44]
*DIR*	*Gossypium hirsutum* (*GhDIR1*)	Promoted the degree of lignification and enhanced *Verticillium dahliae* resistance	[45]
*CHS*	*Zea mays* (*CHS C2*)	Increased lignin content of maize	[11]

*4CL*, 4-coumarate: CoA ligase; *CCR*, cinnamoyl-CoA reductase; *CAD*, cinnamyl alcohol dehydrogenase; *MOMT*: monolignol 4-*O*-methyltransferase; *COMT*, caffeic acid *O*-methyltransferase; *CSE*, caffeoyl shikimate esterase; *CCoAOMT*, caffeoyl-CoA *O*-methyltransferase; *HCT*, hydroxycinnamoyl-CoA shikimate/Quinatehydroxycinnamoyltransferase; *F5H*, ferulate 5-hydroxylase; *POD*, peroxidase; *LAC*, laccase; *ABC*, ATP-binding cassette transporter; *MYB*, v-myb avian myeloblastosis viral oncogene homolog; *MADS*, MADS-box; *HSF*, heat shock factors; *SOD*, superoxide dismutase; *APX*, ascorbate peroxidase; *DIR*, dirigent-like; *CHS*, chalcone synthase.

## References

[1] Ralph J., Lundquist K., Brunow G., Lu F., Kim H., Schatz P.F., Marita J.M., Hatfield R.D., Ralph S.A., Christensen J.H. (2004). Lignins: Natural polymers from oxidative coupling of 4-hydroxyphenyl-propanoids. Phytochem. Rev..

[2] Alejandro S., Lee Y., Tohge T., Sudre D., Osorio S., Park J., Bovet L., Geldner N., Fernie A.R., Martinoia E. (2012). AtABCG29 is a monolignol transporter involved in lignin biosynthesis. Curr. Biol..

[3] Miao Y., Liu C. (2010). ATP-binding cassette-like transporters are involved in the transport of lignin precursors across plasma and vacuolar membranes. Proc. Natl. Acad. Sci. USA.

[4] Bonawitz N.D., Chapple C. (2010). The genetics of lignin biosynthesis: Connecting genotype to phenotype. Annu. Rev. Genet..

[5] Liu C.J., Miao Y.C., Zhang K.W. (2011). Sequestration and transport of lignin monomeric precursors. Molecules.

[6] Ralph J. (2010). Hydroxycinnamates in lignification. Phytochem. Rev..

[7] Del Río J.C., Rencoret J., Prinsen P., Martínez Á.T., Ralph J., Gutiérrez A. (2012). Structural characterization of wheat straw lignin as revealed by analytical pyrolysis, 2D-NMR, and reductive cleavage methods. J. Agric. Food Chem..

[8] Mottiar Y., Vanholme R., Boerjan W., Ralph J., Mansfield S.D. (2016). Designer lignins: Harnessing the plasticity of lignification. Curr. Opin. Biotechnol..

[9] Singh S., Bashri G., Singh A., Prasad S.M. (2016). Regulation of Xenobiotics in Higher Plants: Signalling and Detoxification.

[10] Lan W., Lu F., Regner M., Zhu Y., Rencoret J., Ralph S.A., Zakai U.I., Morreel K., Boerjan W., Ralph J. (2015). Tricin, a flavonoid monomer in monocot lignification. Plant Physiol..

[11] Eloy N., Voorend W., Lan W., Saleme M.L., Cesarino I., Vanholme R., Smith R.A., Goeminne G., Pallidis A., Morreel K. (2017). Silencing chalcone synthase impedes the incorporation of tricin in lignin and increases lignin content. Plant Physiol..

[12] Del Río J.C., Rencoret J., Gutiérrez A., Kim H., Ralph J. (2017). Hydroxystilbenes are monomers in palm fruit endocarp lignins. Plant Physiol..

[13] Schuetz M., Benske A., Smith R.A., Watanabe Y., Tobimatsu Y., Ralph J., Demura T., Ellis B., Samuels A.L. (2014). Laccases direct lignification in the discrete secondary cell wall domains of protoxylem. Plant Physiol..

[14] Ithal N., Recknor J., Nettleton D., Maier T., Baum T.J., Mitchum M.G. (2007). Developmental transcript profiling of cyst nematode feeding cells in soybean roots. Mol. Plant Microbe Interact..

[15] Tripathi S.C., Sayre K.D., Kaul J.N., Narang R.S. (2003). Growth and morphology of spring wheat (*Triticum aestivum* L.) culms and their association with lodging: Effects of genotypes, N levels and ethephon. Field Crop. Res..

[16] Rest B.V.D., Rochange S.F. (2006). Down-regulation of cinnamoyl-CoA reductase in tomato (*Solanum lycopersicum* L.) induces dramatic changes in soluble phenolic pools. J. Exp. Bot..

[17] Shadle G., Chen F., Srinivasa Reddy M.S., Jackson L., Nakashima J., Dixon R.A. (2007). Down-regulation of hydroxycinnamoyl CoA: Shikimate hydroxycinnamoyl transferase in transgenic alfalfa affects lignification, development and forage quality. Phytochemistry.

[18] Derikvand M.M., Sierra J.B., Ruel K., Pollet B., Do C.T., Thévenin J., Buffard D., Jouanin L., Lapierre C. (2008). Redirection of the phenylpropanoid pathway to feruloyl malate in *Arabidopsis* mutants deficient for cinnamoyl-CoA reductase 1. Planta.

[19] Moura J.C.M.S., Bonine C.A.V., Viana J.D.O.F., Dornelas M.C., Mazzafera P. (2010). Abiotic and biotic stresses and changes in the lignin content and composition in plants. J. Integr. Plant Biol..

[20] Vanholme R., Storme V., Vanholme B., Sundin L., Christensen J.H., Goeminne G., Halpin C., Rohde A., Morreel K., Boerjan W. (2012). A systems biology view of responses to lignin biosynthesis perturbations in *Arabidopsis*. Plant Cell.

[21] Huang J., Gu M., Lai Z., Fan B., Shi K., Zhou Y., Yu J., Chen Z. (2010). Functional analysis of the *Arabidopsis PAL* gene family in plant growth, development, and response to environmental stress. Plant Physiol..

[22] Gui J., Shen J., Li L. (2011). Functional characterization of evolutionarily divergent 4-coumarate:coenzyme a ligases in rice. Plant Physiol..

[23] Wagner A., Donaldson L., Kim H., Phillips L., Flint H., Steward D., Torr K., Koch G., Schmitt U., Ralph J. (2009). Suppression of 4-coumarate-CoA ligase in the coniferous gymnosperm *Pinus radiata*. Plant Physiol..

[24] Thevenin J., Pollet B., Letarnec B., Saulnier L., Gissot L., Maiagrondard A., Lapierre C., Jouanin L. (2011). The simultaneous repression of CCR and CAD, two enzymes of the lignin biosynthetic pathway, results in sterility and dwarfism in *Arabidopsis thaliana*. Mol. Plant.

[25] Van Acker R., Déjardin A., Desmet S., Hoengenaert L., Vanholme R., Morreel K., Laurans F., Kim H., Santoro N., Foster C. (2017). Different metabolic routes for coniferaldehyde and sinapaldehyde with CINNAMYL ALCOHOL DEHYDROGENASE1 deficiency. Plant Physiol..

[26] Giordano A., Liu Z., Panter S.N., Dimech A.M., Shang Y., Wijesinghe H., Fulgueras K., Ran Y., Mouradov A., Rochfort S. (2014). Reduced lignin content and altered lignin composition in the warm season forage grass *Paspalum dilatatum* by down-regulation of a Cinnamoyl CoA Reductase Gene. Transgenic Res..

[27] Tu Y., Rochfort S., Liu Z., Ran Y., Griffith M., Badenhorst P., Louie G.V., Bowman M.E., Smith K.F., Noel J.P. (2010). Functional analyses of caffeic acid *O*-methyltransferase and Cinnamoyl-CoA-Reductase genes from perennial ryegrass (*Lolium perenne*). Plant Cell.

[28] Vanholme B., Cesarino I., Goeminne G., Kim H., Marroni F., Van Acker R., Vanholme R., Morreel K., Ivens B., Pinosio S. (2013). Breeding with rare defective alleles (BRDA): A natural *Populus nigr*a *HCT* mutant with modified lignin as a case study. New Phytol..

[29] Coleman H.D., Park J., Nair R., Chapple C., Mansfield S.D. (2008). RNAi-mediated suppression of p-coumaroyl-CoA 3′-hydroxylase in hybrid poplar impacts lignin deposition and soluble secondary metabolism. Proc. Natl. Acad. Sci. USA.

[30] Zhang K., Bhuiya M., Pazo J.R., Miao Y., Kim H., Ralph J., Liu C. (2012). An engineered Monolignol 4-*O*-Methyltransferase depresses lignin biosynthesis and confers novel metabolic capability in *Arabidopsis*. Plant Cell.

[31] Wagner A., Tobimatsu Y., Phillips L., Flint H., Geddes B., Lu F., Ralph J. (2015). Syringyl lignin production in conifers: Proof of concept in a Pine tracheary element system. Proc. Natl. Acad. Sci. USA.

[32] Takeda Y., Koshiba T., Tobimatsu Y., Suzuki S., Murakami S., Yamamura M., Rahman M.M., Takano T., Hattori T., Sakamoto M. (2017). Regulation of CONIFERALDEHYDE 5-HYDROXYLASE expression to modulate cell wall lignin structure in rice. Planta.

[33] Vanholme R., Cesarino I., Rataj K., Xiao Y., Sundin L., Goeminne G., Kim H., Cross J., Morreel K., Araujo P. (2013). Caffeoyl shikimate esterase (CSE) is an enzyme in the lignin biosynthetic pathway in *Arabidopsis*. Science.

[34] Ha C.M., Escamilla-Trevino L., Yarce J.C.S., Kim H., Ralph J., Chen F., Dixon R.A. (2016). An essential role of caffeoyl shikimate esterase in monolignol biosynthesis in *Medicago truncatula*. Plant J..

[35] Saleme M., Cesarino I., Vargas L., Kim H., Vanholme R., Goeminne G., Van R.A., Fonseca F., Pallidis A., Voorend W. (2017). Silencing CAFFEOYL SHIKIMATE ESTERASE affects lignification and improves saccharification. Plant Physiol..

[36] Eudes A., Dutta T., Deng K., Jacquet N., Sinha A., Benites V.T., Eek B., Richel A., Sattler S.E., Northen T.R. (2017). *SbCOMT* (*Bmr12*) is involved in the biosynthesis of tricin-lignin in sorghum. PLoS ONE.

[37] Liu H., Guo Z., Gu F., Ke S., Sun D., Dong S., Liu W., Huang M., Xiao W., Yang G. (2016). 4-Coumarate-CoA ligase-like gene *OsAAE3* negatively mediates the rice blast resistance, floret development and lignin biosynthesis. Front. Plant Sci..

[38] Yang Q., He Y., Kabahuma M., Chaya T., Kelly A., Borrego E., Bian Y., El K.F., Yang L., Teixeira P. (2017). A gene encoding maize caffeoyl-CoA *O*-methyltransferase confers quantitative resistance to multiple pathogens. Nat. Genet..

[39] Herrero J., Fernándezpérez F., Yebra T., Novouzal E., Pomar F., Pedreño M.Á., Cuello J., Guéra A., Estebancarrasco A., Zapata J.M. (2013). Bioinformatic and functional characterization of the basic peroxidase 72 from *Arabidopsis thaliana* involved in lignin biosynthesis. Planta.

[40] Zhao Q., Nakashima J., Chen F., Yin Y., Fu C., Yun J., Shao H., Wang X., Wang Z., Dixon R.A. (2013). LACCASE is necessary and nonredundant with PEROXIDASE for lignin polymerization during vascular development in *Arabidopsis*. Plant Cell.

[41] Guo H., Wang Y., Wang L., Hu P., Wang Y., Jia Y., Zhang C., Zhang Y., Zhang Y., Wang C. (2017). Expression of the MYB transcription factor gene *BplMYB46* affects abiotic stress tolerance and secondary cell wall deposition in *Betula platyphylla*. Plant Biotechnol. J..

[42] Li H., Yang Y., Wang Z., Guo X., Liu F., Jiang J., Liu G. (2016). *BpMADS12* gene role in lignin biosynthesis of *Betula platyphylla* Suk by transcriptome analysis. J. For. Res..

[43] Zeng J.K., Li X., Zhang J., Ge H., Yin X.R., Chen K.S. (2016). Regulation of loquat fruit low temperature response and lignification involves interaction of heat shock factors and genes associated with lignin biosynthesis. Plant Cell Environ..

[44] Shafi A., Chauhan R., Gill T., Swarnkar M.K., Sreenivasulu Y., Kumar S., Kumar N., Shankar R., Ahuja P.S., Singh A.K. (2015). Expression of *SOD* and *APX* genes positively regulates secondary cell wall biosynthesis and promotes plant growth and yield in *Arabidopsis* under salt stress. Plant Mol. Biol..

[45] Tronchet M., Balagué C., Kroj T., Jouanin L., Roby D. (2010). Cinnamyl alcohol dehydrogenases-C and D, key enzymes in lignin biosynthesis, play an essential role in disease resistance in *Arabidopsis*. Mol. Plant Pathol..

[46] Berthet S., Demontcaulet N., Pollet B., Bidzinski P., Cezard L., Bris P.L., Borrega N., Herve J., Blondet E., Balzergue S. (2011). Disruption of LACCASE4 and 17 results in tissue-specific alterations to lignification of *Arabidopsis thaliana* stems. Plant Cell.

[47] Kim Y., Kim C.Y., Song W., Park D., Kwon S., Lee H., Bang J., Kwak S. (2008). Overexpression of sweetpotato swpa4 peroxidase results in increased hydrogen peroxide production and enhances stress tolerance in tobacco. Planta.

[48] Shigeto J., Itoh Y., Hirao S., Ohira K., Fujita K., Tsutsumi Y. (2015). Simultaneously disrupting *AtPrx2*, *AtPrx25* and *AtPrx71* alters lignin content and structure in *Arabidopsis* stem. J. Integr. Plant Biol..

[49] Cosio C., Ranocha P., Francoz E., Burlat V., Zheng Y., Perry S.E., Ripoll J.J., Yanofsky M., Dunand C. (2016). The class III peroxidase PRX17 is a direct target of the MADS-box transcription factor AGAMOUS-LIKE15 (AGL15) and participates in lignified tissue formation. New Phytol..

[50] Wang J., Wang C., Zhu M., Yu Y., Zhang Y., Wei Z. (2008). Generation and characterization of transgenic poplar plants overexpressing a cotton laccase gene. Plant Cell Tissue Organ Cult..

[51] Wang Y., Bouchabke-Coussa O., Lebris P., Antelme S., Soulhat C., Gineau E., Dalmais M., Bendahmane A., Morin H., Mouille G. (2015). LACCASE5 is required for lignification of the *Brachypodium distachyon* Culm. Plant Physiol..

[52] Legay S., Lacombe E., Goicoechea M., Briere C., Seguin A., Mackay J., Grimapettenati J. (2007). Molecular characterization of EgMYB1, a putative transcriptional repressor of the lignin biosynthetic pathway. Plant Sci..

[53] Legay S., Sivadon P., Blervacq A., Pavy N., Baghdady A., Tremblay L., Levasseur C., Ladouce N., Lapierre C., Seguin A. (2010). EgMYB1, an R2R3 MYB transcription factor from *eucalyptus* negatively regulates secondary cell wall formation in *Arabidopsis* and poplar. New Phytol..

[54] Zhong R., Richardson E.A., Ye Z.H. (2007). Two NAC domain transcription factors, SND1 and NST1, function redundantly in regulation of secondary wall synthesis in fibers of *Arabidopsis*. Planta.

[55] Gallego-Giraldo L., Shadle G., Shen H., Barrosrios J., Fresquet C.S., Wang H., Dixon R.A. (2016). Combining enhanced biomass density with reduced lignin level for improved forage quality. Plant Biotechnol. J..

[56] Willis J.D., Smith J.A., Mazarei M., Zhang J., Turner G.B., Decker S.R., Sykes R.W., Poovaiah C.R., Baxter H.L., Mann D.G.J. (2016). Downregulation of a UDP-arabinomutase gene in switchgrass (*Panicum virgatum* L.) results in increased cell wall lignin while reducing arabinose-glycans. Front. Plant Sci..

[57] Fornalé S., Rencoret J., García-Calvo L., Encina A., Rigau J., Gutiérrez A., Del Río J.C., Caparros-Ruiz D. (2017). Changes in cell wall polymers and degradability in maize mutants lacking 3′- and 5′-*O*-Methyltransferases involved in lignin biosynthesis. Plant Cell Physiol..

[58] Barros J., Serk H., Granlund I., Pesquet E. (2015). The cell biology of lignification in higher plants. Ann. Bot..

[59] Bonawitz N.D., Kim J.I., Tobimatsu Y., Ciesielski P.N., Anderson N.A., Ximenes E., Maeda J., Ralph J., Donohoe B.S., Ladisch M. (2014). Disruption of mediator rescues the stunted growth of a lignin-deficient *Arabidopsis* mutant. Nature.

[60] Liljegren S.J., Ditta G.S., Eshed Y., Savidge B., Bowman J.L., Yanofsky M.F. (2000). SHATTERPROOF MADS-box genes control seed dispersal in *Arabidopsis*. Nature.

[61] Liang M., Davis E., Gardner D., Cai X., Wu Y. (2006). Involvement of AtLAC15 in lignin synthesis in seeds and in root elongation of *Arabidopsis*. Planta.

[62] Schilmiller A.L., Stout J., Weng J.K., Humphreys J., Ruegger M.O., Chapple C. (2009). Mutations in the cinnamate 4-hydroxylase gene impact metabolism, growth and development in *Arabidopsis*. Plant J..

[63] Berry P.M., Sterling M., Spink J.H., Baker C.J., Sylvesterbradley R., Mooney S.J., Tams A.R., Ennos A.R. (2004). Understanding and reducing lodging in cereals. Adv. Agron..

[64] Tanaka K., Murata K., Yamazaki M., Onosato K., Miyao A., Hirochika H. (2003). Three distinct rice cellulose synthase catalytic subunit genes required for cellulose synthesis in the secondary wall. Plant Physiol..

[65] Islam M.S., Peng S., Visperas R.M., Ereful N., Bhuiya M.S.U., Julfiquar A.W. (2007). Lodging-related morphological traits of hybrid rice in a tropical irrigated ecosystem. Field Crop. Res..

[66] Zhang B., Zhou Y. (2011). Rice brittleness mutants: A way to open the ‘black box’ of monocot cell wall biosynthesis. J. Integr. Plant Biol..

[67] Peng D., Chen X., Yin Y., Lu K., Yang W., Tang Y., Wang Z. (2014). Lodging resistance of winter wheat (*Triticum aestivum* L.): Lignin accumulation and its related enzymes activities due to the application of paclobutrazol or gibberellin acid. Field Crop. Res..

[68] Zheng M., Jin C., Shi Y., Li Y., Yin Y., Yang D., Luo Y., Pang D., Xu X., Li W. (2017). Manipulation of lignin metabolism by plant densities and its relationship with lodging resistance in wheat. Sci. Rep..

[69] Hu D., Liu X.B., She H.Z., Gao Z., Ruan R.W., Wu D.Q., Yi Z.L. (2017). The lignin synthesis related genes and lodging resistance of *Fagopyrum esculentum*. Biol. Plant..

[70] Dorairaj D., Ismail M.R., Sinniah U.R., Tan K.B. (2017). Influence of silicon on growth, yield, and lodging resistance of MR219, a lowland rice of Malaysia. J. Plant Nutr..

[71] Jie K., Sun Y., Zhou M., Zhang P., Zuo Q., Wu J., Zhou G. (2016). The effect of nitrogen application and planting density on the radiation use efficiency and the stem lignin metabolism in rapeseed (*Brassica napus* L.). Field Crop. Res..

[72] Zhang W., Wu L., Ding Y., Xiong Y., Wu X., Fei W., Li G., Liu Z., She T., Ding C. (2017). Nitrogen fertilizer application affects lodging resistance by altering secondary cell wall synthesis in japonica rice (*Oryza sativa*). J. Plant Res..

[73] Wang C., Hu D., Liu X., She H., Ruan R., Yang H., Yi Z., Wu D. (2015). Effects of uniconazole on the lignin metabolism and lodging resistance of culm in common buckwheat (*Fagopyrum esculentum* M.). Field Crop. Res..

[74] Kong L., Sun M., Wang F., Liu J., Feng B., Si J., Zhang B., Li S., Li H. (2014). Effects of high NH_4_^+^ on K^+^ uptake, culm mechanical strength and grain filling in wheat. Front. Plant Sci..

[75] Schützendübel A., Polle A. (2002). Plant responses to abiotic stresses: Heavy metal-induced oxidative stress and protection by mycorrhization. J. Exp. Bot..

[76] Liu Q., Zheng L., He F., Zhao F.J., Shen Z., Zheng L. (2015). Transcriptional and physiological analyses identify a regulatory role for hydrogen peroxide in the lignin biosynthesis of copper-stressed rice roots. Plant Soil.

[77] Jannoey P., Pongprasert W., Lumyong S., Roytrakul S., Nomura M. (2015). Comparative proteomic analysis of two rice cultivars (*Oryza sativa* L.) contrasting in brown planthopper (BPH) stress resistance. Plant Omics.

[78] Santiago R., Barrosrios J., Malvar R.A. (2013). Impact of cell wall composition on maize resistance to pests and diseases. Int. J. Mol. Sci..

[79] Duan C., Yu J., Bai J., Zhu Z., Wang X. (2014). Induced defense responses in rice plants against small brown planthopper infestation. Crop J..

[80] Jannoey P., Channei D., Kotcharerk J., Pongprasert W., Nomura M. (2017). Expression analysis of genes related to rice resistance against brown planthopper, *Nilaparvata lugens*. Rice Sci..

[81] Wang Y., Sheng L., Zhang H., Du X., An C., Xia X., Chen F., Jiang J., Chen S. (2017). *CmMYB19* over-expression improves aphid tolerance in Chrysanthemum by promoting lignin synthesis. Int. J. Mol. Sci..

[82] Fujimoto T., Mizukubo T., Abe H., Seo S. (2015). Sclareol induces plant resistance to root-knot nematode partially through ethylene-dependent enhancement of lignin accumulation. Mol. Plant Microbe Interact..

[83] Tianpei X., Li D., Qiu P., Luo J., Zhu Y., Li S. (2015). Scorpion peptide LqhIT2 activates phenylpropanoid pathways via jasmonate to increase rice resistance to rice leafrollers. Plant Sci..

[84] Zhang S., Yang Q., Ma R. (2007). *Erwinia carotovora* ssp. *carotovora* infection induced “defense lignin” accumulation and lignin biosynthetic gene expression in chinese cabbage (*Brassica rapa* L. ssp. *pekinensis*). J. Integr. Plant Biol..

[85] Karkonen A., Koutaniemi S. (2010). Lignin biosynthesis studies in plant tissue cultures. J. Integr. Plant Biol..

[86] Miedes E., Vanholme R., Boerjan W., Molina A. (2014). The role of the secondary cell wall in plant resistance to pathogens. Front. Plant Sci..

[87] Ma Q.H., Zhu H.H., Han J.Q. (2017). Wheat ROP proteins modulate defense response through lignin metabolism. Plant Sci..

[88] Mandal S., Kar I., Mukherjee A.K., Acharya P. (2013). Elicitor-induced defense responses in *Solanum lycopersicum* against *Ralstonia solanacearum*. Sci. World J..

[89] Shi H., Liu Z., Zhu L., Zhang C., Chen Y., Zhou Y., Li F., Li X. (2012). Overexpression of cotton (*Gossypium hirsutum*) dirigent1 gene enhances lignification that blocks the spread of *Verticillium dahliae*. Acta Biochim. Biophys. Sin..

[90] Wang G.F., He Y., Strauch R., Olukolu B.A., Nielsen D., Li X., Balint-Kurti P.J. (2015). Maize homologs of Hydroxycinnamoyltransferase, a key enzyme in lignin biosynthesis, bind the nucleotide binding leucine-rich repeat Rp1 proteins to modulate the defense response. Plant Physiol..

[91] Chen G., Liu Y., Wang R., Zhang J., Owens G. (2013). Cadmium adsorption by willow root: The role of cell walls and their subfractions. Environ. Sci. Pollut. Res. Int..

[92] Dalcorso G., Farinati S., Furini A. (2010). Regulatory networks of cadmium stress in plants. Plant Signal. Behav..

[93] Demirbas A. (2004). Adsorption of lead and cadmium ions in aqueous solutions onto modified lignin from alkali glycerol delignication. J. Hazard. Mater..

[94] Guo X., Zhang S., Shan X.Q. (2008). Adsorption of metal ions on lignin. J. Hazard. Mater..

[95] Bhuiyan N.H., Liu W., Liu G., Selvaraj G., Wei Y., King J. (2007). Transcriptional regulation of genes involved in the pathways of biosynthesis and supply of methyl units in response to powdery mildew attack and abiotic stresses in wheat. Plant Mol. Biol..

[96] Kováčik J., Bačkor M. (2007). Phenylalanine ammonia-lyase and phenolic compounds in chamomile tolerance to cadmium and copper excess. Water Air Soil Pollut..

[97] Tahara K., Norisada M., Hogetsu T., Kojima K. (2005). Aluminum tolerance and aluminum-induced deposition of callose and lignin in the root tips of *Melaleuca* and *Eucalyptus* species. J. For. Res.

[98] Lima J.D., Mazzafera P., Moraes W.D.S., Silva R.B.D. (2009). Chá: Aspectos relacionados à qualidade e perspectivas tea: Aspects related to the quality and prospects. Ciênc. Rural.

[99] Ghanati F., Morita A., Yokota H. (2005). Deposition of suberin in roots of soybean induced by excess boron. Plant Sci..

[100] Mao C., Yi K., Yang L., Zheng B., Wu Y., Liu F., Wu P. (2004). Identification of aluminium-regulated genes by cDNA-AFLP in rice (*Oryza sativa* L.): Aluminium-regulated genes for the metabolism of cell wall components. J. Exp. Bot..

[101] Gao L., Peng K., Chen Y., Wang G., Shen Z. (2012). Roles of apoplastic peroxidases, laccases, and lignification in the manganese tolerance of hyperaccumulator *Phytolacca americana*. Acta Physiol. Plant.

[102] Lin C.C., Chen L.M., Liu Z.H. (2005). Rapid effect of copper on lignin biosynthesis in soybean roots. Plant Sci..

[103] Van de Mortel J.E., Villanueva L.A., Schat H., Kwekkeboom J., Coughlan S., Moerland P.D., Van Themaat E.V.L., Koornneef M., Aarts M.G.M. (2006). Large expression differences in genes for iron and zinc homeostasis, stress response, and lignin biosynthesis distinguish roots of *Arabidopsisthaliana* and the related metal hyperaccumulator *Thlaspi caerulescens*. Plant Physiol..

[104] Yang Y.J., Cheng L.M., Liu Z.H. (2007). Rapid effect of cadmium on lignin biosynthesis in soybean roots. Plant Sci..

[105] Akgül M., Çöpür Y., Temiz S. (2007). A comparison of kraft and kraft-sodium borohydrate brutia pine pulps. Build. Environ..

[106] Ederli L., Reale L., Ferranti F., Pasqualini S. (2004). Responses induced by high concentration of cadmium in Phragmites australis roots. Physiol. Plant..

[107] Van de Mortel J.E., Schat H., Moerland P.D., Ver Loren van Themaat E., Ent S.V.D., Blankestijn H., Ghandilyan A., Tsiatsiani S., Aarts M.G.M. (2008). Expression differences for genes involved in lignin, glutathione and sulphate metabolism in response to cadmium in *Arabidopsis thaliana* and the related Zn/Cd-hyperaccumulator *Thlaspi caerulescens*. Plant Cell Environ..

[108] Liu Q., Le L., Wang X., Shen Z., Zheng L. (2017). Comprehensive analysis of rice laccase gene (*OsLAC*) family and ectopic expression of *OsLAC10* enhances tolerance to copper stress in *Arabidopsis*. Int. J. Mol. Sci..

[109] Feng J., Jia W., Lv S., Bao H., Miao F., Zhang X., Wang J., Li J., Li D., Zhu C. (2017). Comparative transcriptome combined with morpho-physiological analyses revealed key factors for differential cadmium accumulation in two contrasting sweet sorghum genotypes. Plant Biotechnol. J..

[110] Ahsan N., Nakamura T., Komatsu S. (2012). Differential responses of microsomal proteins and metabolites in two contrasting cadmium (Cd)-accumulating soybean cultivars under Cd stress. Amino Acids.

[111] Chaves M.M., Flexas J., Pinheiro C. (2009). Photosynthesis under drought and salt stress: Regulation mechanisms from whole plant to cell. Ann. Bot..

[112] Agarwal P.K., Shukla P.S., Gupta K., Jha B. (2012). Bioengineering for salinity tolerance in plants: State of the art. Mol. Biotechnol..

[113] Monties B., Fukushima K., Hofrichter M., Steinbuchel A. (2001). Occurrence, Function and Biosynthesis of Lignins. Biopolymers. Lignin, Humic Substances and Coal.

[114] Mourasobczak J., Souza U., Mazzafera P. (2011). Drought stress and changes in the lignin content and composition in *Eucalyptus*. BMC Proc..

[115] Hu Y., Li W.C., Xu Y.Q., Li G.J., Liao Y., Fu F.L. (2009). Differential expression of candidate genes for lignin biosynthesis under drought stress in maize leaves. J. Appl. Genet..

[116] Fan L., Linker R., Gepstein S., Tanimoto E., Yamamoto R., Neumann P.M. (2006). Progressive inhibition by water deficit of cell wall extensibility and growth along the elongation zone of maize roots is related to increased lignin metabolism and progressive stelar accumulation of wall phenolics. Plant Physiol..

[117] Srivastava S., Vishwakarma R.K., Arafat Y.A., Gupta S.K., Khan B.M. (2015). Abiotic stress induces change in Cinnamoyl CoA Reductase (CCR) protein abundance and lignin deposition in developing seedlings of *Leucaena leucocephala*. Physiol. Mol. Biol. Plants.

[118] Yamaguchi M., Valliyodan B., Zhang J., Lenoble M.E., Yu O., Rogers E.E., Nguyen H.T., Sharp R.E. (2010). Regulation of growth response to water stress in the soybean primary root. I. Proteomic analysis reveals region-specific regulation of phenylpropanoid metabolism and control of free iron in the elongation zone. Plant Cell Environ..

[119] Park S.C., Kim Y.H., Jeong J.C., Kim C.Y., Lee H.S., Bang J.W., Kwak S.S. (2011). Sweetpotato late embryogenesis abundant 14 (*IbLEA14*) gene influences lignification and increases osmotic- and salt stress-tolerance of transgenic calli. Planta.

[120] Kováčik J., Grúz J., Klejdus B., Štork F., Marchiosi R., Ferrarese Filho O. (2010). Lignification and related parameters in copper-exposed *Matricaria chamomilla* roots: Role of H_2_O_2_ and NO in this process. Plant Sci..

[121] Christensen J.H., Christensen O.B. (2007). A summary of the PRUDENCE model projections of changes in European climate by the end of this century. Clim. Chang..

[122] Bita C.E., Gerats T. (2013). Plant tolerance to high temperature in a changing environment: Scientific fundamentals and production of heat stress-tolerant crops. Front. Plant Sci..

[123] Guy C., Kaplan F., Kopka J., Selbig J., Hincha D.K. (2008). Metabolomics of temperature stress. Physiol. Plant..

[124] Wei H., Dhanaraj A.L., Arora R., Rowland L.J., Fu Y., Sun L. (2006). Identification of cold acclimation-responsive Rhododendron genes for lipid metabolism, membrane transport and lignin biosynthesis: Importance of moderately abundant ESTs in genomic studies. Plant Cell Environ..

[125] Gindl W., Grabner M., Wimmer R. (2000). The influence of temperature on latewood lignin content in treeline Norway spruce compared with maximum density and ring width. Trees.

[126] Yun Z., Gao H., Ping L., Liu S., Tao L., Shuai J., Qiang X., Xu J., Cheng Y., Deng X. (2013). Comparative proteomic and metabolomic profiling of citrus fruit with enhancement of disease resistance by postharvest heat treatment. BMC Plant Biol..

[127] Zhao Q., Tobimatsu Y., Zhou R., Pattathil S., Gallego-Giraldo L., Fu C., Jackson L.A., Hahn M.G., Kim H., Chen F. (2013). Loss of function of cinnamyl alcohol dehydrogenase 1 leads to unconventional lignin and a temperature-sensitive growth defect in *Medicago truncatula*. Proc. Natl. Acad. Sci. USA.

[128] Gellerstedt G., Henriksson G. (2008). Lignins: Major Sources, Structure and Properties. Monomers, Polymers and Composites from Renewable Resources.

[129] Chen F., Tobimatsu Y., Havkinfrenkel D., Dixon R.A., Ralph J. (2012). A polymer of caffeyl alcohol in plant seeds. Proc. Natl. Acad. Sci. USA.

[130] Constant S., Wienk H.L.J., Frissen A.E., Peinder P.D., Boelens R., Es D.S.V., Grisel R.J.H., Weckhuysen B.M., Huijgen W.J.J., Gosselink R.J.A. (2016). New insights into the structure and composition of technical lignins: A comparative characterisation study. Green Chem..

[131] Lupoi J.S., Singh S., Parthasarathi R., Simmons B.A., Henry R.J. (2015). Recent innovations in analytical methods for the qualitative and quantitative assessment of lignin. Renew. Sustain. Energy Rev..

[132] Ragauskas A.J., Williams C.K., Davison B.H., Britovsek G., Cairney J., Eckert C.A., Frederick W.J., Hallett J.P., Leak D.J., Liotta C.L. (2006). The path forward for biofuels and biomaterials. Science.

[133] Monteil-Rivera F., Phuong M., Ye M., Halasz A., Hawari J. (2013). Isolation and characterization of herbaceous lignins for applications in biomaterials. Ind. Crop. Prod..

[134] Azadfar M., Gao A.H., Bule M.V., Chen S. (2015). Structural characterization of lignin: A potential source of antioxidants guaiacol and 4-vinylguaiacol. Int. J. Biol. Macromol..

[135] Vinardell M.P., Mitjans M. (2017). Lignins and their derivatives with beneficial effects on human health. Int. J. Mol. Sci..

